# “Can You Hear Me? Can Anybody Pretty Please Validate [Me]?”: The Long Shadow of Separation and Liminality in Military Physicians’ Remediation

**DOI:** 10.5334/pme.2463

**Published:** 2026-09-11

**Authors:** Tasha R. Wyatt, Witzard Seide, Emily Scarlett, Rachel Chamberlin, Jessica Bunin

**Affiliations:** 1 Department of Health Professions Education, Uniformed Services University, Bethesda, MD, USA; 2 Independent Scholar, USA; 3 School of Nursing, University of Texas, San Antonio, TX, USA; 4 Department of Anesthesiology, Henry Jackson Foundation, Uniformed Services University of the Health Sciences, Bethesda, MD, USA; 5 Independent Scholar

## Abstract

**Introduction:**

Much of the current literature on remediation centers on procedural steps, due process, and the implementation of model programs. However, these studies frequently omit the critical perspective of the learners undergoing the process, which can reveal hidden forces that shape medical students’ experiences.

**Methods:**

We conducted narrative interviews with 15 practicing physicians who remediated as medical students 5–15 years ago. Data were analyzed by examining plot, characters, and event sequences. Upon identifying a pattern of stages, we applied van Gennep’s three-phase framework of rites of passage: *separation, liminality, and aggregation*.

**Results:**

Remediation remained a vivid, salient experience for participants, characterized by disorientation. Separation was marked by strained community interactions and symbolic “parting.” The liminal phase involved isolation and ambiguity while awaiting institutional decisions. Notably, aggregation, reintegration into the medical community, was achieved by few; more than half of the participants still feel excluded or marginalized years later.

**Discussion:**

Remediation is a harrowing process with long-lasting professional consequences. While all participants experienced separation and liminality, the failure to achieve aggregation highlights the “long shadow” remediation casts over a remediated physicians’ career. We suggest that remediation committees consider providing a ritual specific to the reintegration phase to ensure learners feel successfully folded back into the professional community.

## Introduction

Remediation of medical students is defined as, ‘*the act of facilitating a correction for trainees who started out on the journey toward becoming a physician but have moved off course*’ [1 (p.xvii)]. Struggling learners are remediated for a variety of reasons, which include maladjustment in their transition to medical school, time management, over-reliance on passive learning strategies, and inadequate content and knowledge preparation [2,3,4]. Such struggles are normal for individuals transitioning into new settings, but often come as a shock to medical students who have a long history of high performance [5]. At the heart of remediation practices is the profession’s concern around issues of competency and whether the learner will function well as a practicing physician. Thus, remediation committees serve as micro-cultures in that they are not merely administrative bodies but are self-contained worlds where members possess their own distinct language, unspoken rules, class dynamics, and mechanisms that mirror and reinforce the broader society and medical profession. Thus, they serve as gatekeepers holding sovereign power over entry into the profession.

Despite recent institutional efforts to develop supportive remediation [1,5], this gatekeeping process inherently triggers intense student emotions including feelings of guilt, embarrassment, and shame [6], emotions that arise with the aftermath of failure. In other cases, feelings of anger, fear, anxiety, and sadness have been reported [6]. A broader perspective frames these learners as entering a career inflection point where they are no longer just students on a learning path but now judged in terms of their clinical trustworthiness. By viewing remediation not merely as a bureaucratic or academic hurdle, but as a high-stakes cultural ritual, this process can be framed as transitional space where their past academic identities and future professional survival is called into question by elders who judge them as capable or incapable of being a physician [7].

Much of the current literature on remediation centers on procedural steps, due process, and the implementation of model programs [7]. However, these studies frequently omit the critical perspective of the learners undergoing the process, which can reveal hidden forces that shape medical students’ experiences. In a departure from previous research on this topic, this study captures the narratives of practicing physicians who were remediated as medical students during their undergraduate medical training within the last 15 years. We chose to capture narratives after a significant period of time had elapsed to see what these physicians recalled about the process, what impacted them most, and the long-term consequences of this event, all in hopes of improving the remediation process. Specifically, we focused on a specific type of remediation, which our institution calls deceleration; the process of holding students back a year. Deceleration is arguably one of the most detrimental types of remediation compared to others (i.e. repeating a course) because it has long-term consequences that extend beyond the learners’ time in medical training. This study was guided by the following research question: *How do practicing physicians retrospectively narrate their past experiences with remediation (when they were medical students) and its effects on their career?*

## Methods

This qualitative study recruited military physicians from our institution’s Long-Term Career Outcomes Study (LTCOS) to participate. LTCOS houses records of all the medical students who have graduated dating back to 1976 and provided a means to track graduates into their current clinical positions. LTCOS also provided up to date contact information for all graduates so that longitudinal research on a physician’s career can be conducted. In this study, we narrowed our focus to those military physicians who had undergone remediation within the past 15 years, meaning we focused on individuals who matriculated between 2006 and 2016. Like civilian physicians, these individuals are practicing in various clinical settings, such as in-patient clinics, hospitals, etc., however their patient population is purely active-duty military members and their families. Unlike civilian physicians, each of them has a dual identity as both a doctor and military officer in the United States military.

Remediation in this study context refers to students who were held back for an additional year of medical school. We focused on this form of remediation (as opposed to those who merely failed an exam) because of the extended timeline participants experience in the process, and the potential impact remediation might have on their life course and careers. Additionally, we chose to set the date range of 5–15 years because we wanted enough time to have passed for participants to reflect on their remediation experience and its impact, but not too much time that they could no longer recall the experience. In total, 61 physicians fit our criteria for having been remediated within the last 15 years, with most having done so within the last 10. For context, prior to 2011, the school had a model of 2 years of preclinical (i.e. classroom) time followed by 2 years of clinical (i.e. clerkship) time. After 2011, the curriculum shifted to 18 months preclinical and 30 months clinical. The majority of our participants would have experienced the former. We emailed all 61 physicians twice, using both their work and personal emails, and 15 agreed to participate. Table 1 outlines the reasons why they were remediated as well as what participants perceived triggered the remediation process. Table 2 provides a description of participant characteristics.

**Table 1:** Reasons and Triggers for Deceleration.


REASON FOR DECELERATION	AMOUNT (%)

Low grades	3 (20)

Poor adjustment to coursework	5 (33)

Step 1 issues	3 (20)

Failed exam or module	3 (20)

Professionalism	1 (7)

**ISSUES PARTICIPANTS FELT CONTRIBUTED TO DECELERATION**	**AMOUNT (%)**

Personal stressors	9 (60)

Mental health issues and personal stressors	1 (7)

Undiagnosed sleep disorder	1 (7)

Cultural struggles	1 (7)

None identified	3 (7)


**Table 2:** Participant Characteristics.


GENDER	AMOUNT (%)

Male	8 (53)

Female	7 (47)

**RACE/ETHNICITY**	**AMOUNT (%)**

White	4 (27)

unstated	8 (53)

other/multiracial	3 (20)

**DECELERATION YEAR**	**AMOUNT (%)**

2007	1 (7)

2008	3 (20)

2009	3 (20)

2010	1 (7)

2011	1 (7)

2012	2 (13)

2013	1 (7)

2014	2 (13)

2017	1 (7)


Using an online platform, we interviewed 13 of the 15 participants for 45–60 minutes on their experiences of remediation. These semi-structured interviews included questions, such as “*Put yourself back into the timeframe of when you experienced remediation. Describe each of the steps that you went through in that process*” and “*Now that some time has passed since you experienced remediation, how do you feel about the experience*?” See Appendix A for a complete list of interview questions. Two participants submitted textual responses because their location was hidden for security purposes and they could not access video platforms; we treated their written data like the interviews and included them in our analytical process. As data collection progressed, each interview was transcribed and discussed by the research team. These discussions focused on similarities and differences across participants and helped to construct an overarching understanding of their remediation experience. Although we reached a point where no new conceptual insights were apparent by the 12^th^ participant, we decided to continue data collection to be certain of our decision, ending with a final count of 15 participants, 13 of which were interviewed and two (2) who had submitted written reflections.

We then formally analyzed these data using narrative analysis [8]; a family of analytical approaches that focuses on how participants tell stories of their lives. We chose narrative analysis because stories are the way individuals create meaning about various events, which has potential to provide critical insight into the human condition [9]. This first level of analysis helped us see various elements of the participants’ stories including the overall plot being narrated, who the main and supporting characters were, the order of events that took place, and the outcomes at each point in the story. In the analysis, we noticed an emerging pattern that showed stages in remediation. As a result, we consulted the ‘transitions theory’ literature [10,11] for a way to describe their experiences and found numerous references to rites of passage [12,13,14]. Finding this framework helpful, in the second level of analysis, we analyzed participants’ narratives through van Geenep’s [15] three phases that describe *rites of passage* (i.e. *separation, liminality* and *aggregation*) to capture the overall experiences of these students.

*Rites of passage* focus on individuals’ *transitions* and take the form of ceremonies or acts which accompany “any change in social state, age, place or life cycle stage [16 (p.41)].” Van Gennep [15] describes rites of passage (i.e. transitions from one social position to another) in terms of three phases: *separation, liminality*, and *aggregation*. *Separation* occurs when an individual experiences detachment from their daily activities, which starts to signal that the individual’s relationship to the community has shifted. This phase is often anxiety provoking because the world they knew is no longer the world they are in. *Liminality* is marked by what Turner [13] describes as being ‘betwixt and between;’ a brief period of chaos whereby individuals enter into an interstitial space within the community’s social structure. Others have described this phase as being unclean and polluted [17]. This phase of being ‘here nor there’ is extremely confusing and wrought with emotions of instability. The final phase is *aggregation* and is comprised of *reintegration* and a welcoming back into the community as a changed individual.

We chose a *rites of* passage framework because remediating students start out as members of the community, but once they are identified as struggling for any number of reasons (e.g. weak transition to medical school, poor time management, over-reliance on passive learning strategies, inadequate preparation, etc. [2,3,4]), their relationship to and with the community shifts. Kalet [1] and Ellaway [18] have both argued that undergoing remediation is a *liminal* experience because it marks a major inflection point whereby the learner is judged to be capable of being a physician (or not) by a governing body (i.e. remediation committee), in which they have little influence. Once we analyzed the data from a rites of passage framework, we used constant comparative analysis [19] to compare each participants’ experience with the others and cross-case analysis [19] to find patterns, similarities and differences to identify participants’ experiences of remediation.

We intentionally created a diverse research team with experiences in remediation as well as sensitivity to cultural rituals. TW studies the ways that power is used in medical education to shape and coerce students to be the *right* kind of physician [20]. WS is a pediatrician who at the time worked in GME as a faculty mentor to medical students facing challenges during medical school. RC is a medical anthropologist with a background in behavioral and community health and works at the intersections of culture and medicine. ES was our research assistant, and JB held a senior leadership position around issues of community and belonging. None of us have been remediated ourselves. Rather our interest in this topic stems from institutional leaders and educators who have interacted with those who have been remediated and the experience they have shared with us. This study was approved by Uniformed Services University under IRB#: DBS.2022.442.

## Results

Over a decade after participants remediated, their experience of remediation remained a salient experience in these physicians’ careers. Participants could easily recall vivid memories and feelings of uncertainty and disorientation as they transitioned through *separation, liminality*, and *aggregation*, but the transition through the three phases was not uniform or complete; for some participants, they remained unclean and beyond acceptable social categories [17]. Participants described *separation* as comprising strained interactions with their professional community and heightened awareness of symbols communicating a parting. In the *liminal* state, they experienced isolation and ambiguity while their fate was decided; it was only ameliorated when community members or others in remediation reached out to check on their well-being. The final phase, *aggregation*, which was not offered by the remediation committee, was experienced by only a few. Without a structured process to reintegrate these remediated students back into the professional community, many described that they still do not feel reintegrated into the profession 5–15 years after their remediation experience. The results are organized by each of the three phases in rites of passage with the understanding that these phases overlap as individuals transition through them.

### Separation: *“I was not offered grace … [and] my life’s falling apart”*

As participants were told of their need to appear before the remediation committee, they described feelings of *separation*. One participant described having to write a statement to the committee explaining what happened, but they felt like they did not have “enough time to give an accurate accounting of what was going on” (P13). This participant indicated they were “pretty embarrassed about having to [remediate] in the first place so, I wasn’t really looking into kind of the reasons behind [it]” (P13). Others were aware of the external stressors they were experiencing and, in these cases, went into the meeting actively requesting remediation, as this participant shared:

After talking with [my mentors, we] came to the conclusion that I should [remediate] and I obviously agreed with it. I [asked] the board for [remediation] so that I can have an extra year to spread out all of the rest of my rotations, manage my divorce, and manage how I would maximize my time with my daughter. (P15)

As participants narrated their experiences of deceleration, which was 5–15 years earlier, they began their narratives by describing the symbolism they noticed in the room where decisions on who would be remediated took place. In this physical space, they described their first experience parting with the medical community. It came in material form, in which the participants detailed the arrangement of furniture including what the room looked and felt like, and where leaders sat in relation to them. In these descriptions, there were frequent references to a long U-shaped table where the student sat on one side and the rest of Committee members sat on the other, as one described, “You are sitting at the edge of the table …[at] this whole U-shaped room of older mostly Caucasian men” (P6). Others could recall approximately how many people were in the room, a recollection that remained because they were all staring at them from the other side: “There was literally like 20–25 of the leadership in a horseshoe” (P2). In every description of the physical layout, participants found this arrangement symbolic of who had the power and who did not: “It was completely intimidating being the one person at the end of the table and the other people deciding your fate” (p14). This physical arrangement made a lasting impression in terms of the overall experience, and many confessed still being affected by the experiences in ways that continually surprise them, “I’m definitely still traumatized from it… The power difference between them and you, that was really traumatizing” (P4). Others described it as “probably one of the scariest things I’ve ever done in my life” (P2).

Participants described their interactions with Committee interactions as contributory to the *separation* process with several narrating they felt malicious intent stemming from the Committee. For example, despite their understanding that the Committee was meant to support them in their journey back to training, this participant indicated some members were out to get them: “I think people wanted to see me knocked down” (P1). Others described it feeling “Kafka-esque” (P11) or “an opportunity [for the Committee] to express their superiority over you” (p10) and that the whole process made them feel small and insignificant: “It made me feel like I wasn’t worth the effort from anyone in administration” (P3). Others indicated they felt “kind of embarrassed … going to talk to these guys [that] obviously got through med school” (P13) recognizing that there was a clear distinction between them and the participant that further aggravated the separation of the two. They indicated that this separation was made worse when the opening statements were made because the Committee’s interactions made them feel as if they had committed a crime and what they had done was unforgivable:

I didn’t get the sense it was redeemable. I felt like I was clawing through and trying to create a pathway through which they could give me grace. I was not offered grace. … my life’s falling apart… It didn’t really feel like they were trying to help me to find that way to redemption. (P10)

Those who do not have specific memories of being in the room still narrated similar feelings. Even without the specific memory of presenting their case, participants felt a sense of inevitability around their fate. In other words, once this rite of passage was initiated, there was no going back:

I recall being told that I could give a statement. I feel like I did, but I also remember feeling like it made no difference. I felt like their minds were made up already that I was either [to] remediate or get kicked out. (P3)

In the *separation* phase, participants narrated feeling disoriented because the Committee did not explain *how* participants could engage in this process or even *why* they were being remediated. For example, when one participant had failed an exam, no one asked about what precipitated the failure: “They didn’t have questions. [They didn’t ask] what happened. They didn’t seek to clarify anything. [I was left wondering] what I had done” (P5). Another participant was being considered for remediation because of professionalism issues, but no one explained what she had done wrong, “I was in my first rotation. I was failed for professionalism and nobody explained [it] to me” (P11). Others found that the information they received was generic and unhelpful. For example, they would receive advice such as, “study and fix yourself” (P3). However, such comments did not help with safe passage, as this one explained: “Clearly, if I knew how to study, I wouldn’t be in the situation!” (P3). These strained interactions combined with the symbolism participants felt in the physical location of the room where the verdict was made, marked not only a separation in the terms of their daily activities (e.g. repeating the year), but also in their emotional connection to the medical community who signaled a parting of ways.

### Liminality: *“Nobody’s looking out for you”*

Entering the state of *liminality* began when participants presented themselves and their case to the Committee. Several indicated how infuriating it was to feel the Committee’s expectation that they bare their entire private lives in front of strangers to be allowed to continue with training. For example, this participant narrated her isolation, “I remember reading my letter and crying. I don’t cry very often… [I was] just feeling just so sad about it. [When] I looked up, I remember thinking there was no empathy” (P6). Participants indicated other students who had remediated did not need to present their case to the Committee because their situation was seen as less serious, a difference that contributed to a sense of instability in their membership to the profession. Some described feeling they had to play specific roles just so the Committee would recommend remediation over disenrollment, which they characterized as a permanent banishing from the community. In one case, the participant felt she was expected to play on the trope of a helpless woman:

They want to hear a sad weakened little girl who’s crying before them begging them to use their powerful position …[in this] anointed spot at this table. They want to be able to decide whether they’re going to give you the privilege of their forgiveness… I was totally and completely caught off guard by that. (P1)

She decided not to cry when she came before the Committee yet was told later, by sympathetic Committee members, that by not crying, the Committee felt she appeared arrogant and lacking in humility. This left her extremely confused because she had deliberately chosen not to cry aligning with what she felt was a different expectation from the Committee. In recalling the experience, she shared,

It took every fiber in my being not to cry because I thought that I’m a military officer, and I’m sitting in a committee having screwed up, [and] I damn well better hold it together and be strong because that’s the professional thing to do. (P1)

In her case, confusion came from not knowing what role she was supposed to play. Was she supposed to cry and show insight into her situation or was she supposed to be a strong military officer who can handle tough situations? This *liminal* space, in which she was caught between being a struggling medical student (on the brink of being separated from the community) and being a military officer (who was still a part of the community) was incredibly disorienting.

Throughout this phase, participants also described feeling isolated. One recalled “feeling unsafe… like nobody’s looking out for you” (P4). Others described feeling completely on their own, a “persona non grata” (P2). This created feelings that as a medical student, you become a person “no one wants to associate with because [they] haven’t decided what to do with you yet” (P2). As a result of this isolation, participants felt it was up to them to fix the situation, “It felt very much like I was figuring it out on my own” (P14). Douglas [17] describes this liminal phase as being temporarily outcast and throughout the duration of the rite, they are treated as marginal beings. In several cases, participants wanted merely to be validated as humans who were going through a challenging time in their lives. In our interview, one participant pretended she was talking to the Committee, saying to them, “Can you hear me? Can anybody pretty please validate that I’m not just a piece of s*** that needs to just get out of your way and disappear?” (P10). Others described wishing someone would have reached out and taken a “human perspective of ‘How are you doing? What are you doing?” (P8) to just check in to make sure they felt that someone was on their side. Many narrated stories about major events, such as match day where their liminality was on display. As they explained:

[The class of] 2015, filled with the people I knew and cared about, matched before me, and [the class of] 2016 didn’t care enough about me to include me. I sharply remember sobbing on my way home from my 2015 friends matching because I was in such a s**** limbo. This, above all else, emotionally haunts me to this day. (P3)

When faculty members outside of the Committee checked in with the students these interactions were healing and helped to mitigate feelings of isolation. For example, one participant described, “There were people who saw the personal aspect of what I was going through … I felt their compassion and …that was important” (P8). In some cases, faculty members helped students fill knowledge gaps, as such as in this situation, “The admin had sat down with me and [said] you’re in trouble and this is what we’re [going to do]” (P4), which brought comfort because “she was the first one that took an interest in how I was doing” (P4). Other faculty members helped guide them through the unknowns, “She one of my instructors … I really liked her and had a good relationship with her. She was kind of my point person … [helping me] navigate and manage everything” (P15). In other cases, participants were able to receive support from their peers, thus reducing their feelings of isolation, “I felt support from not only the staff but also the students during [remediation] and this allowed me to continue to move forward and successfully graduate” (P12). These peers brought them what was needed in this phase, which was “a little bit of camaraderie” (P13).

### Aggregation: *“Still, there’s shame and fear about people knowing”*

Participants unanimously described a lack of *aggregation* and *reintegration* back into the professional community. This lack of ritual appears to have had vastly different long-term consequences. Some hardly think about this time period anymore: “I honestly don’t give it much thought” (P10) and “This was like a blip on the radar for me” (P1). Whereas others described it as a learning experience, something they appreciate much more now: “It was a stressful time, but I learned a tremendous amount” (P7). In some cases, participants would use the same breath to describe their remediation experience as both traumatic and helpful because they felt they successfully made it through the process. For example, this one spoke to the complexity around going through this process: “[It was] an absolutely horrible experience … but, I am stronger now for it [because it] forced me to do some growing” (P10). Others narrated they did not mind the extra year of training: “It hasn’t affected me negatively… other than maybe one extra [year of] your time” (P6), describing it as “academic failures [that] were isolated episodes,” and even going so far as to say, “I gained confidence in my classmates and more importantly, confidence in myself” (P12).

Others were less positive about the experience and complained it “upset my career timeline” (P5) describing negative feelings, such as “I’m a little bitter still” (P11) or “I lost years of my life in terms of career progression. … When I hear the words 2012, which was my graduating year, that’s a little triggering for me” (P12). Despite the one participant who gained confidence, more than half of the participants indicated that remediation had decreased their confidence as a provider, a feeling that follows them to this day. For example, this participant describes how remediation is not just a mark on his CV or transcripts, he feels as if it is a stain against him as a person, which he cannot shake: “While it is just an academic mark, I think it’s linked to my identity” (P14) signaling that they cannot shake the feelings aroused during this time, and continue to relive the experience even years later, as if he is stained, dirty, and unacceptable. For example, this participant described the effect remediation had on him:

It’s still something that I think about. I don’t know why, but it was a tough time. I still struggle with the emotional aspect of it, more so the feelings I had; what I went through during that time. Not that I have bad feelings towards other people, but more my own perception of myself. … I remember always feeling that people will see me as less. (8)

Without a clear ritual or demarcation that they had been reintegrated into the profession, they described experiencing physical integration without emotional integration, which continued to haunt them. The lack of ritual brought up reoccurring feelings of shame and fear, and that somehow being remediated will come back and wreak havoc on them: “I still have that little bit of fear that it’ll come back and haunt me, or threaten my credentials and my livelihood” (P10). At the time, many did not want their colleagues to know when they were going through it or had done so in the past. One explained that even now, as a practicing physician,

I still don’t openly share that I was remediated in med school. I still have fear surrounding that if someone knows, somehow, they’ll think differently of me professionally. If someone knows, I won’t be selected for whatever position. …Staying on my record, 15 years later, having proven myself as a clinician and leader in all the things I’ve done, still, there’s shame and fear about people knowing. (P14)

Interestingly, one of the participants has since joined the remediation Committee and serves as a member adjudicating the fate of other struggling students. She shared that those on the Committee do not fully grasp how much power they have; it is hidden. However, for those subordinated in the system, the Committee’s power is the only thing they can see, which detracts from the work that needs to be done:

Now when I look back, because I was on that Committee a couple years ago when I was at [the Institution], I didn’t see [it]. When you’re in the position of power, you don’t see that at all … but something about being a student in that position really does [affect you]. (P4)

## Discussion

This study explored the experiences of remediation among military physicians through the lens of rites of passage, a process that has existed for centuries and is thought to be occurring everywhere around the world as individuals transition from one state to another [13,15]. While many studies have been conducted on remediation, researchers have yet to analyze their data using a rites of passage framing to understand what learners go through in this transition. Published studies seem to privilege institutional perspectives over students’, which has left an incomplete picture on the structural and educational effectiveness of remediation. By reframing remediation as a rite of passage, not only do we slow down this process for a deeper analysis in ways that other studies have struggled [21,22], reframing also allows for critique and thus the possibility for change.

One of the major findings in the current study speaks to the emotional turmoil students experience, thus expanding the work of Mills et al., [23] which demonstrated that this transition is harrowing and filled with long-lasting emotions that extend beyond the time they are in medical training [22,24]. In the current study, as soon as participants were notified that something was amiss, they sensed a parting or what others writing about rites of passage have described as “a generalized social bond that [will soon] cease to be [13 (p.360)].” They described looking for symbolism in their environment to substantiate a shifting relationship with the medical community that foreshadowed the new arrangement with the institution and the profession itself. Bourgeois-Law et al. [25] highlighted a possible reason why physicians feel this. Those who remediate are generally perceived by the medical community as different, somehow both ‘one of us’ and ‘not one of us’ simultaneously. This may in part be because those responsible for remediation frequently shift between conceptualizing the process as both an aspect of students’ educational continuum and sometimes as a regulatory process [26]. The lack of clarity on what remediation is and how the community frames it sends mixed messages to those going through it. Therefore, without clarity on what remediation is, an aspect of learning or regulatory process, participants seem to look for symbols to make sense of what they are experiencing. This study describes the kinds of symbols students noticed in their environment and the effect it had on them.

Participants then entered a *liminal* phase as they transitioned into a holding pattern on their journey to becoming a physician. Hayashi et al. [27] noted that remediating Generation Z students were initially resistant to the medical profession during the remediation process, experiencing conflict between various identities both within and outside of medical school. Although the participants in the current study were not all Generation Z physicians, they too experienced a sense of ungrounding. Those who study rites of passage call it “elud[ing] or slip[ping] through the network of classifications that normally locate states and positions in cultural space [13 (p.359)].” Research shows that this is an emotionally dark and isolating part of the path: “Liminality is frequently likened to death, to being in the womb, to invisibility [13 (p.359)].” In this study, participants described that the only light they received were small gestures made by faculty members or other remediating students who reached out, thus signaling that the professional community was still available to them, albeit in a constrained way. This finding reveals the importance of regularly checking in on students in this liminal phase, and paying attention to how they are doing as individuals not just their academic progress [28].

Most interesting, because there was no clearly defined *aggregation* phase, participants could not *reintegrate* into the professional community as evidenced in their continued emotional struggle 5–15 years later. While clearly all the participants successfully completed the remediation process, and have gone on to be successful physicians, and obviously rejoined the medical community, the third phase in the rites of passage framework is unfinished. The results suggest that while the Committee and the institution may feel they have welcomed the student back into the profession upon completion of remediation, to the remediated student, their professional identity and self-image have been irreversibly changed. The rites of passage framework suggests that without a clear signal from the Committee that they are welcomed back in, the first two phases of *separation* and *liminality* continue to cast a long shadow even after the formal remediation process is complete.

This long shadow is akin to what is known as being *haunted*, a concept that originated in hauntology and spectral studies [29], and has recently moved into medical and health professions education [30]. It has since been applied only to medical education curriculum, but has promise in explaining other experiences. Simply, the *haunted curriculum* acknowledges that there is trauma in medical education curricula that comes through in various spaces, curricular content, and forms of absence. It suggests, “a lurking presence [that] ought to be reckoned with history that rings in the present.” (p.270) Belling [31] indicates that doctors and other health professionals feel ‘haunted’ when they have unresolved sorrow or regret around clinical issues, such as the death of a patient, or sociocultural issues in their community, such as racism and slavery, both of which can emerge within curricular design. Haunting is both an emotional and epistemological experience that directly connects one’s past professional trauma and future failure.

In the context of this study, these remediated physicians may be thought of as ‘one of us’ [25] by the Committee, but internally, their identification as a community member in the medical profession was far from it. This conflict in identification may even be internalized in the participants themselves. It’s unclear if this tension lies between their conscious thought and subconscious emotions or just comes up when they are given reminders of remediation. Regardless, this finding suggests that Committee members should consider incorporating a ritual of sorts marking the students’ completion of their transition through the final stage.

According to Douglas [17], acknowledgement of this final stage is critical: “With no rite of aggregation which can definitely assign him to a new position he remains in the margins, with other people who are similar credited with unreliability, unteachability, and all the wrong social attitudes.” (p.2) We suspect that no matter how successful these physicians are, they will never feel whole again without an explicit gesture from those who cast them out initially. Therefore, one idea for medical schools to consider is to use restorative justice [32,33] to help individuals reintegrate into the community. While the practice frequently evokes associations with punishment and wrongdoing that require repair, it actually emphasizes healing, relational, and reintegrative potential. Restorative justice brings those who were harmed and those who committed the harm together to acknowledge that regardless of a failure, the individual is still a part of the community. This ceremony is incredibly useful when an individual has committed a harm, such as theft, cheating, or violating other community norms, but the ritual helps with the emotional aspects of reintegration into a community, which others have noted is critical to the process [21,34].

While our study uses a novel analytical lens to understand the experiences of military physicians who underwent remediation, we must note a few limitations. First, contexts shape remediation practices [7], and this study was conducted in a military medical school which includes an additional form of hierarchy (e.g. military rank). Unlike civilian medical schools, these medical students are also military officers and thus need to adhere to power differentials and chains of command, regardless of the setting. Participants alluded to issues of power and subordination in the remediation process, as well as confusion around which identity to bring forth (i.e. military officer vs. struggling medical student) as they presented themselves before the Committee. Interestingly, despite the participants’ struggle with their dual identity, they did not mention needing to be reintegrated into the military. This is because military physicians can be reassigned to other duties but will remain in the military. The student’s position in the military is never in jeopardy in remediation. However, having a dual hierarchy raises questions around the complexity of navigating two hierarchical systems. Military physicians need clear guidance in such situations, so they know exactly what is expected of them. Future research should explore military physicians’ navigation process, including when they make decisions to foreground one identity over the other, their reasons for doing so, and any potential consequences.

Additionally, there may have been a potential selection bias among those who responded to our invitation. Although we got a mix of participant experiences, those who reflected enough to create a cohesive narrative may be the ones who responded to our call. Also, the military is a closed system, which has implications for the long-term effects of those who decelerate. Military physicians cannot simply move away and start their practice elsewhere as an anonymous professional; they interact with those they have trained with, thus potentially heightening their feelings of not being able to escape the experience and feeling the weight of not having a ritual for reintegration.

In conclusion, this study examined the long-term experiences of practicing military physicians who underwent remediation as medical students 5 to 15 years ago. The initial separation they felt from the medical community, compounded by the liminal chaos of losing a stable professional identity, triggered severe emotional distress. Crucially, because their institution lacked a formal ritual to mark their successful reintegration into the profession, the trauma of remediation was deeply cemented. As a result, even years into successful independent practice, these physicians continue to carry a profound, lingering sense of being professional outsiders.

## Disclaimer

This work was prepared by employees of the US Government as part of their official duties and therefore is in the public domain. The opinions and assertions expressed herein are those of the author(s) and do not necessarily reflect the official policy or position of the Uniformed Services University or the Department of War.

## Additional File

The additional file for this article can be found as follows:

10.5334/pme.2463.s1 Appendix A. Interview Questions.

## References

[1] Kalet A, Chou C. Remediation in Medical Education. Springer; 2014. DOI: 10.1007/978-1-4614-9025-8

[2] Dolan S, Mallott DB, Emery JA. Passive learning: a marker for the academically at risk. Med Teach. Nov 2002;24(6):648–649. DOI: 10.1080/01421590216082 12623462

[3] Burns ER. Learning syndromes afflicting beginning medical students: identification and treatment—reflections after forty years of teaching. Med Teach. May 2006;28(3):230–233. DOI: 10.1080/01421590600632920 16753720

[4] Paul G, Hinman G, Dottl S, Passon J. Academic development: a survey of academic difficulties experienced by medical students and support services provided. Teach Learn Med. Jul 2009;21(3):254–260. DOI: 10.1080/10401330903021041 20183347

[5] Winston K, Van der Vlueten C, Scherpbeir A. At-risk medical students: implications of students’ voice for the theory and practice of remediation. Medical Education. 2010;44(10):1038–1047. DOI: 10.1111/j.1365-2923.2010.03759.x 20880373

[6] Jay R, Hagan P, Madan C, Patel R. A phenomenological exploration of the feedback experience of medical students after summative exam failure. BMC Medical Education. 2023/12/08 2023;23(1):930. DOI: 10.1186/s12909-023-04892-z 38066543 PMC10709976

[7] Kalet A, Chou C, Ellaway R. To fail is human: Remediating remediation in medical education. Perspectives on Medical Education. 2017;6(418–424). DOI: 10.1007/S40037-017-0385-6 PMC5732108 29071550

[8] Herman L, Vervaeck B. Handbook of Narrative Analysis. University of Nebraska Press; 2019. DOI: 10.2307/j.ctvr43mhw

[9] Sarbin T. Narrative Psychology: The Storied Nature of Human Conduct. Praeger Publishers/Greenwood Publishing Group; 1986.

[10] Schlossberg N. A model for analyzing human adaptation to transition. The Counseling Psychologist. 1981;9(2):1–18. DOI: 10.1177/001100008100900202

[11] Schlossberg N. The challenge of change: The transition model and its applications. Journal of Employment Counseling. 2011;48(4):159–162. DOI: 10.1002/j.2161-1920.2011.tb01102.x

[12] Turner V. Betwixt and between: The liminal period in rites de passage. University of Washington Press; 1964.

[13] Turner V. Liminality and Communitas. The Ritual Process: Structure and Anti-Structure. Aldine Publishing; 1969.

[14] Hensley N. Passage and pivot points: Experience and education as rites of passage. Journal of Curriculum Theorizing. 2019;34(4):27–37. DOI: 10.63997/jct.v34i4.718

[15] van Gennep A. The rights of passage (Trans. by Monika B Vizedom and Gabrielle L Caffee. Routledge and Kegan Paul; 1960. DOI: 10.7208/chicago/9780226027180.001.0001

[16] Bell B. The rites of passage and outdoor education: Critical concerns for effective programming. The Journal of Experiential Education. 2003;26(1):41–50. DOI: 10.1177/105382590302600107

[17] Douglas M. Powers and Dangers. Purity and Danger: An analysis of the concepts of pollution and taboo. Routledge; 1996. pp. 1–12.

[18] Ellaway R, Kalet A. Situating remediation: accommodating success and failure in medical education systems. Academic Medicine. 2018. DOI: 10.1097/ACM.0000000000001855 28767496

[19] Strauss A, Corbin J. Basics of qualitative research: Techniques and procedures for developing grounded theory. 2nd ed. Sage; 1998.

[20] Frost H, Regehr G. “I AM a doctor”: Negotiating the discourses of standardization and diversity in professional identity construction. Academic Medicine. 2013;88(10):1–8. DOI: 10.1097/ACM.0b013e3182a34b05 23969361

[21] Bourgeois-Law G, Regehr G, Teunissen P, Varpio L. Strangers in a strange land: The experience of physicians undergoing remediation. Medical Education. 2021;56(6):670–679. DOI: 10.1111/medu.14736 35080035

[22] McLeod K, Woodward-Kron R, Rashid P, Archer J, Nestel D. “I’m on an island”: A qualitative study of underperforming surgical trainee perspectives on remediation. The American Journal of Surgery. 2024;234:11–16. DOI: 10.1016/j.amjsurg.2024.01.033 38350749

[23] Mills L, Stenfors T, Duffy M, et al. “When You’re in It, It Feels Like It’s Everything”: Medical students’ experience of failure and remediation in the United States and the Netherlands. Academic Medicine. 2024;99:1254–1259. DOI: 10.1097/ACM.0000000000005845 39137260

[24] Mills L, Boscardin C, Joyce E, ten Cate O, O’Sullivan P. Emotion in remediation: A scoping review of the medical education literature. Medical Education. 2021;55(12):1350–1362. DOI: 10.1111/medu.14605 34355413

[25] Bourgeois-Law G, Teunissen P, Varpio L, Regehr G. Attitudes Towards Physicians Requiring Remediation: One-of-Us or Not-Like-Us? Medical Education. 2019;94:S36–S41. DOI: 10.1097/ACM.0000000000002896 31365392

[26] Bourgeois-Law G, Varpio L, Regehr G, Teunissen PW. Education or regulation? Exploring our underlying conceptualisations of remediation for practising physicians. Medical Education. 2019;53:276–284. DOI: 10.1111/medu.13745 30345526

[27] Hayashi M, Karouji Y, Nishiya K. Ambivalent professional identity of early remedial medical students from Generation Z: A qualitative study. BMC Medical Education. 2022;22(501). DOI: 10.1186/s12909-022-03583-5 PMC9237971 35761249

[28] Price T, Wong G, Withers L, et al. Optimising the delivery of remediation programmes for doctors: A realist review. Medical Education. 2021;55:995–1010. DOI: 10.1111/medu.14528 33772829

[29] Derrida J. Specters of Marx: The state of debt, the work of mourning, and the new international. Routledge; 1994.

[30] Ansari D, Jain N, Tucker C, Karlin J. When I say … haunted curriculum. Medical Education. 2024. DOI: 10.1111/medu.15537 PMC11789835 39410913

[31] Belling C. Haunted Doctors. Perspectives in Biology and Medicine. 63(3):466–479. DOI: 10.1353/pbm.2020.0034 33416620

[32] Acosta D, Karp D. Restorative justice as the Rx for mistreatment in academic medicine: Applications to consider for learners, faculty, and staff. Academic Medicine. 2018;93(3):354–356. DOI: 10.1097/ACM.0000000000002037 29087964

[33] Sawin G, Klasson C, Kaplan S, et al. Scoping review of restorative justice in academics and medicine: A powerful tool for justice, equity, diversity, and inclusion. Health Equity. 2023;7(1):663–675. DOI: 10.1089/heq.2023.0071 37786530 PMC10541936

[34] Chung H, Chung N, Harper J. Betwixt and between: An exercise in work identity and transition. Management Teaching Review. 2024. DOI: 10.1177/23792981241267761

