# Supplementary material for: “Can You Hear Me? Can Anybody Pretty Please Validate [Me]?”: The Long Shadow of Separation and Liminality in Military Physicians’ Remediation
Source: Perspect Med Educ. 2026 Sep 11;15(1):819–29. doi: 10.5334/pme.2463 (PMC13565038; doi:10.5334/pme.2463)
Supplement: Supplementary file 1 [file pme-15-1-2463-s1.pdf]

Interview Questions:

1. Put yourself back into the timeframe of when you experienced deceleration. Describe each of the steps that you went through in that process. For example, can you describe:
    a. When in your training did you experience deceleration?
    b. What precipitated it?
    c. Who was involved?
    d. What were the steps you went through?
2. If you feel comfortable sharing, at the time, what was going on in your personal life that might have affected you and/or led to deceleration?
3. How did it affect you in terms of your life, studies, and relationships?
4. What do you wish USU would have known at that time?
5. What do you wish USU would have done differently at that time?
6. Now that some time has passed since you experienced deceleration, how do you feel about the experience?
7. In what ways, if any, did this process play a role in your trajectory/professional choices?
8. Why did you want to do this interview?

Age
Gender
Year of Remediation
Race
Ethnicity
